# Innovative Techniques and Challenges in Securing Endotracheal Tubes Among Patients With Facial Hair: A Scoping Review

**DOI:** 10.1002/hsr2.72721

**Published:** 2026-06-30

**Authors:** Shaqayeq Taghizadeh, Kimia Khonakdar, Shaghayegh Rezaeekia, Seyed Abolfazl Hosseini, Alireza Babajani

**Affiliations:** ^1^ Department of Anesthesiology, Faculty of Paramedical Sciences Guilan University of Medical Sciences Rasht Iran; ^2^ Department of Anesthesiology, School of Allied Medical Sciences Mazandaran University of Medical Sciences Sari Iran; ^3^ Department of Operating Room and Anesthesiology, Faculty of Paramedical Sciences Guilan University of Medical Sciences Rasht Iran; ^4^ Department of Anesthesiology, School of Allied Medical Sciences Kashan University of Medical Sciences Kashan Iran; ^5^ Department of Anesthesiology, School of Allied Medical Sciences Alborz University of Medical Sciences Karaj Iran

**Keywords:** airway, endotracheal tube, facial hair, scoping review, securing

## Abstract

**Background and Aims:**

Securing endotracheal tubes in patients with facial hair who refuse to shave remains a significant clinical challenge for anesthesiologists and intensive care specialists. This scoping review aims to identify and evaluate innovative endotracheal tube fixation techniques in these patients.

**Methods:**

A comprehensive search was conducted in PubMed, Scopus, Web of Science, Embase, Online Library, Cochrane Library, Google Scholar, and Gray Literature Sources, following PRISMA‐ScR guidelines. The search focused on articles published between 1990 and April 2026. Eligible articles comprised case reports, case series, letters, editorials, reviews, and clinical trials related to endotracheal tube fixation methods in patients with facial hair. Studies without relevant fixation methods or without full text were excluded. Titles, abstracts, and full texts were independently screened by two senior anesthesiologists, and discrepancies were resolved by a third reviewer. A descriptive thematic analysis was conducted to identify patterns and systematically categorize fixation techniques.

**Results:**

From an initial pool of 47 studies, seven met the inclusion criteria: six case reports/letters and one randomized clinical trial. Three innovative endotracheal tube fixation categories were identified: facial coverings using Tegaderm, Dynaplast, or modified serum bottles; remote securement via straps or tapes positioned away from the face; and mustache‐based stabilization involving ligation of the endotracheal tube to the patient's elongated mustache. Reported advantages included facial hair preservation, reduced jugular vein pressure, minimal surgical field disruption, wide materials accessibility, avoidance of adhesive‐related reactions, and cost‐effectiveness. However, identified limitations included lack of direct comparisons with conventional fixation techniques, limited generalizability due to anecdotal evidence bases, and insufficient evaluation in diverse surgical positions.

**Conclusion:**

Current evidence regarding standard methods for securing endotracheal tubes in patients with facial hair is limited, and long‐term safety and efficacy are insufficiently evaluated. Future randomized controlled trials for comparing these innovative approaches with conventional methods are required.

## 1 Introduction

Airway management is a fundamental aspect of both general anesthesia and critical care. Among the available techniques, endotracheal intubation is widely considered the gold standard for securing the airway in most surgical procedures and in patients requiring mechanical ventilation [1, 2]. After the correct placement of the endotracheal tube is confirmed, secure fixation is critical to prevent tube displacement or inadvertent extubating [3, 4]. Inadequate tube securement may lead to serious complications, including hypoxia and cardiopulmonary arrest [5].

Adhesive tape is currently the most used method for securing endotracheal tubes [6]. However, proper adhesion requires a clean, dry, and hair‐free skin surface. Therefore, this approach is less effective in patients with facial hair, such as beards or mustaches, who elect not to shave due to cultural, religious, or personal reasons. In these patients, the use of adhesive tape is associated with a heightened risk of accidental tube dislodgement [7].

To address this limitation, several alternative strategies have been developed. Tube bandages and holders are commonly used, but these devices present notable disadvantages, including compression of the jugular veins and a consequent increase in intracranial pressure, making them less suitable for neurosurgical procedures [8]. An additional approach involves directly suturing the endotracheal tube to anatomical structures around the oral region, such as the upper lip. While this technique can provide strong tube fixation, it is invasive and may cause local tissue injury, infection, and aesthetic concerns, which have constrained its use in clinical practice [9].

Currently, there is no standardized protocol for fixing these tubes in patients with facial hair. As a result, healthcare providers often use innovative but insufficiently evaluated techniques. This study aims to identify and analyze reported methods for endotracheal tube fixation in patients with facial hair, systematically describe these techniques, summarize their outcomes, and highlight gaps in the available evidence to guide future research.

## 2 Materials and Methods

### 2.1 Study Design

This study was conducted in accordance with the PRISMA‐ScR guidelines (Supplementary File 1) and aimed to examine methods for endotracheal tube fixation in patients with facial hair, including their reported advantages and limitations. The protocol of this scoping review was registered in the PROSPERO database (Registration No: 1139909).

### 2.2 Study Questions

1. What evidence is available concerning the methods used to secure endotracheal tubes in patients with facial hair?
2. What advantages have been reported for these methods?
3. What limitations have been noted regarding these methods?

The Population, Concept, and Context (PCC) framework for this study is summarized in Table 1.

**TABLE 1:** PCC for scoping review.

Population	Patients with facial hair admitted to hospital units, including intensive care, operating rooms, and emergency departments, may require endotracheal intubation.
Concept	Methods for securing endotracheal tubes to prevent displacement in patients with facial hair include various techniques and materials, such as adhesives, sutures, binders, and retention devices.
Context	Clinical experience suggests that this approach may be advantageous across multiple hospital settings, including nursing, anesthesia, intensive care, and respiratory therapy. Furthermore, it has demonstrated utility in studies addressing airway management in patients with particular anatomical features, such as facial hair, which can pose unique challenges. Its application is not limited by geographic or temporal constraints.

### 2.3 Search Strategy

An initial literature search was conducted across multiple databases, including PubMed, Web of Science, Scopus, Cochrane Library, Embase, and Gray Literature Databases such as OpenGrey, as well as the Google Scholar search engine. The search focused on studies published between 1990 and April 2026. A comprehensive and systematic search strategy was developed to identify relevant studies. The search terms included a combination of keywords and Boolean operators as follows: (“way” or “method” or “method” or “technique” or “approach”) and (“secure” or “maintain*” or “secure” or “fix” or “fixate” or “fixate”) and (“intratracheal intubation*” or “intratracheal intubation*” or “intratracheal intubation”) and (“facial hair” or “beard*” or “mustache” or “mustache” or “hirsutism”). It should be noted that the search strategy is described separately for the major databases listed in Supplementary File 2.

### 2.4 Inclusion Criteria

This review included studies examining various methods of endotracheal tube fixation in patients with facial hair across all age groups. Eligible publications comprised editorials, case reports, case series, letters, commentaries, reviews, original research articles, and clinical trials published in English.

### 2.5 Exclusion Criteria

Studies that specifically focused on endotracheal tube fixation methods in patients without facial hair, as well as those assessing fixation techniques for other airway devices, such as laryngeal mask airways, were excluded. Animal studies were also not included in the analysis. Furthermore, publications that were not in article format, such as books, blogs, and similar sources, were excluded. Additionally, studies lacking full‐text access, which prevented an evaluation of the implementation, advantages, and limitations of the methods used, were also excluded from this review.

### 2.6 Study Protocol

After applying the search strategy and retrieving relevant articles, study selection was conducted independently by two senior anesthesiologists. First, titles and abstracts were screened, followed by full‐text review of eligible articles, and discrepancies were resolved by a third reviewer who made the final decision. After completing the selection of articles, relevant data were extracted using a standardized data extraction table. For articles where the full text was unavailable or more clarification was required, the corresponding authors were contacted via email to obtain the complete manuscript or any additional information whenever possible.

### 2.7 Critical Appraisal

A critical appraisal of the included studies was conducted to assess the methodological quality of the available evidence. Case reports and letters were evaluated using the Joanna Briggs Institute (JBI) critical appraisal checklist for case reports [10]. This checklist includes eight items addressing the clarity of patient characteristics, clinical history, diagnostic methods, intervention, outcomes, adverse events, and key lessons.

The randomized controlled trial included in this review was assessed using the Cochrane Risk of Bias tool 2 (ROB 2) [11], which evaluates five domains: bias arising from the randomization process, deviations from intended interventions, missing outcome data, measurement of the outcome, and selection of the reported results.

### 2.8 Statistical Analysis

Data were extracted using a standardized data extraction form developed by the research team and implemented in Microsoft Excel C6 capturing study characteristics (author, year, and country), study design, sample size, description of fixation methods, reported advantages, and identified limitations. Given that the available evidence consisted predominantly of case reports and descriptive studies, quantitative synthesis and inferential statistical analyses were not appropriate; therefore, no statistical tests were performed. Instead, a descriptive thematic analysis was conducted to identify patterns and systematically categorize fixation techniques, their advantages, and associated limitations.

## 3 Results

### 3.1 Description of the Selected Studies

**FIGURE 1:**
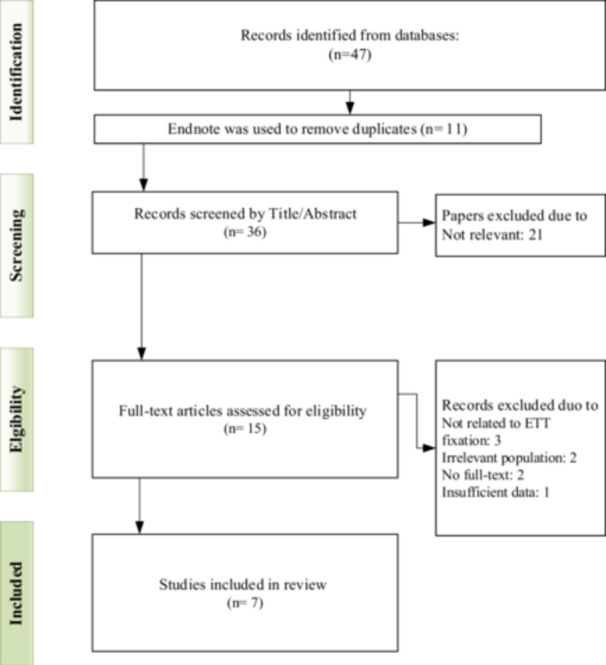
PRISMA 2020 flow diagram of article selection.

This scoping review followed a structured methodological framework. The study selection process is summarized in Figure 1. The initial search identified 47 potentially relevant studies. After removing duplicates and ineligible records and completing title/abstract screening and full‐text assessment, seven studies met the inclusion criteria and were included in the final synthesis. A total of approximately 906 patients were represented across the included studies. The characteristics of the selected studies, as well as the identified endotracheal tube fixation methods and their associated advantages and limitations, are presented in Table 2.

**TABLE 2:** Summary of extraction articles.

Author, Year, and Country	Type of study	Sample size	How to implement the method	Advantages	Limitations
Khorasani et al. 1996, United States (Chicago, Illinois) [12]	Letter/case report	1	In this method, a long mustache (> 1 cm) is used to fix the ETT. The patient's mustache is tightly wrapped around the endotracheal tube, and the endotracheal tube remains in place without the need for adhesive tape on the face or neck.	No neck compression; no facial adhesive; suitable for allergic or edentulous patients (lacking adequate upper jaw/teeth support).	Possible 1–2 cm ETT displacement; the cuff should be positioned 3–4 cm below the vocal cords; Not suitable for airway‐area surgery, non‐supine positions, or long‐term ventilation.
Kamalipour et al. 2003, Iran [13]	Randomized clinical trial	900	The method used a synthetic leather mask with a central hole for ETT passage; the inner surface was made of anti‐allergic thread. The mask was attached to a circular occipital pad with four Velcro straps, and the ETT was secured to the mask using adhesive tape without skin contact.	No skin contact (prevents allergic reactions); avoids jugular compression; no tube displacement; sterilizable and reusable; compatible with laryngoscopy/suction; Suitable for > 2 h anesthesia; better performance than adhesive or cotton tape; comparative data with other fixation methods.	The availability and affordability of this method have not been stated.
			Patients were divided into three groups (mask, adhesive, and neck bandage), and parameters were evaluated 15–120 min after fixation.		
Hooda et al. 2010, India [14]	Letter/case report	1	A 35 × 35 cm “eye” cut from Steri‐Drape. It extends from the forehead to the upper chest, covering the beard. The airway and tracheal tube protrude through the “eye.” Two strips of Dynaplast were placed over the Steri‐Drape to secure the tube.	No shaving; no jugular pressure; no need for specialized equipment; no interference with cervical spine surgical sites; suitable for adhesive‐allergic patients, low‐cost, usable in lateral/prone positions.	Anecdotal evidence with limited generalizability and no comparative data. During extubation, improper removal may pull facial hair; wetting the cloth beforehand reduces this risk.
Agarwal et al. 2011, India [15]	Letter/case report	1	A 500 mL IV plastic bottle is cut into a rectangular sheet. A central groove (equal to the ETT diameter) and two side holes are made, ensuring sufficient distance from edges to prevent tearing. After intubation, the tube is temporarily fixed with a dressing, then placed in the groove and secured to the plastic using adhesive. Finally, the entire assembly (plastic + tube) is fixed to the patient's head with a dressing passed through the side holes.	Provides smooth surface for adhesion; avoids jugular compression; maintains visibility; low‐cost and easily available.	Anecdotal evidence with limited generalizability and no comparative data, requires advance preparation and careful trimming to avoid sharp plastic edges.
Kajal et al. 2015, India [16]	Case report	1	The ETT is first closed at the mouth corner with a gauze bandage. One end of the bandage passes under the axilla (with a protective pad) and is secured to the back of the neck; the opposite end is brought from the other side and tied together posteriorly. Finally, the tube is wrapped with adhesive and fixed to the bridge of the nose to counteract downward traction.	No tension on the tracheal tube, no facial/occipital interference, reduced extubation risk, no jugular pressure, suitable for prone surgeries; simple, low‐cost, no special expertise	Anecdotal evidence with limited generalizability and no comparative data may cause underarm discomfort without adequate padding.
Brahma et al. 2020, India [9]	Case report	1	Non‐adhesive paper tape is wrapped around the ETT and secured at the back of the head. A plastic ring from an IV fluid set is used to stabilize the tube and is attached posteriorly, and the whole assembly is anchored to the operating table with a strap to ensure stability during lateral cranial surgery.	Simple, inexpensive, and accessible; prevents tube displacement; avoids jugular compression; ideal for cranial surgeries in lateral/occipital positions	Anecdotal evidence with limited generalizability and no comparative data.
Singh et al. 2022, India [17]	Letter/case report	1	After intubation, apply a Tegaderm dressing over the beard. Wrap two Dynaplast strips around the ETT and attach them to the Tegaderm. At the end of surgery, remove the dressing easily using an alcohol‐based hand rub.	No shaving; quick, simple, and inexpensive; prevents jugular compression; maintains access for neck/back surgeries; allows skin monitoring; painless removal.	Anecdotal evidence with limited generalizability and no comparative data.

### 3.2 Study Characteristics

The seven included studies exhibited considerable heterogeneity in design and provenance. Four were published as letters to the editor or case reports [12, 14, 15, 17], while two were dedicated case reports [9, 16]. Only one study adopted a higher level of methodological rigor, comprising a randomized clinical trial [13]. Geographically, the majority originated from India (*n* = 5; [9, 14, 15, 16, 17]), with the remainder from Iran (*n* = 1; [13]), and the United States (*n* = 1; [12]).

### 3.3 Critical Appraisal

The methodological quality of the included studies was assessed using the JBI checklist for case reports and the ROB 2 tool for the randomized controlled trial. In the case reports, the intervention was clearly described in all studies, and most reported patient demographic characteristics and clinical conditions. However, patient history presented as a timeline and diagnostic assessments were not reported in any of the case reports. Post‐intervention outcomes were variably reported, and adverse events were not reported in any of the case reports. All studies provided a clear takeaway message. The randomized controlled trial showed some concerns across most ROB 2 domains, with low risk of bias in outcome measurement. The detailed results of the critical appraisal are presented in Supplementary File 3.

### 3.4 Synthesis of Findings

Thematic analysis of the included studies yielded four principal themes, encompassing several subthemes. These themes encompassed: (1) challenges associated with endotracheal tube (ETT) fixation in patients with facial hair; (2) innovative fixation techniques; (3) advantages of these approaches; and (4) complications and limitations.

### 3.5 Challenges in Endotracheal Tube Fixation for Patients With Facial Hair

Across the included studies, three predominant subthemes emerged regarding the difficulties in securing endotracheal tubes (ETTs) in patients with facial hair. First, adhesive tapes exhibited poor adherence owing to interference from facial hair [9, 14, 15, 16]. Second, neck bandages were contraindicated due to the risk of jugular vein compression, which may compromise venous return and elevate intracranial pressure [9, 12, 13, 14, 15, 16, 17]. Third, such bandages posed challenges in head and neck surgical contexts by obstructing procedural access [9, 13, 14, 17]. Furthermore, one study highlighted the potential for spinal cord injury associated with neck bandages in trauma patients [12].

### 3.6 Innovative Endotracheal Tube Fixation Techniques

**FIGURE 2:**
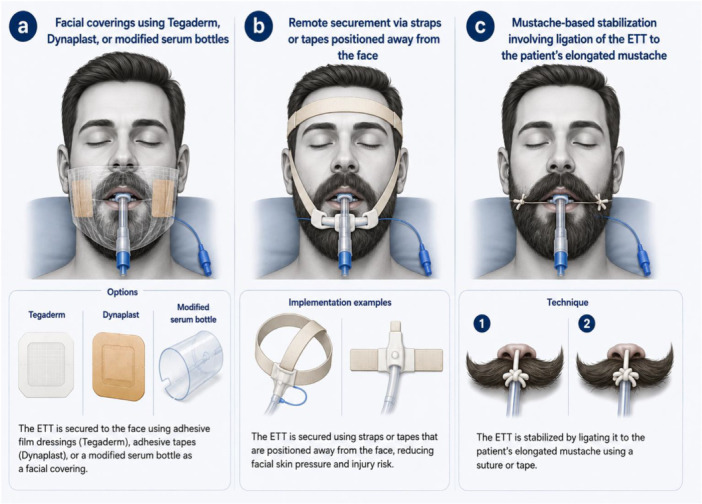
Innovative endotracheal tube fixation techniques (a) facial covering, (b) remote securement, (c) mustache‐based stabilization. “Figure generated with assistance from ChatGPT (OpenAI).”

Thematic synthesis revealed three principal subthemes about innovative fixation strategies (Figure 2). First, the creation of a facial covering represented the most prevalent technique across the included studies [14, 15, 17]. This approach preserves the patient's facial hair while augmenting adhesive tape efficacy through the application of a protective facial barrier. Coverings were fashioned from diverse materials, including repurposed plastic serum bottles [15]. Alternatively, pliable dressings such as Tegaderm and Dynaplast [14, 17]. Second, remote fixation methods entailed securing the endotracheal tube at distal sites to circumvent facial hair interference. Securing straps were affixed via axillary routes, posterior head encircling, the surgical table, or the facemask [9, 16, 17]. Third, mustache‐based fixation involved ligating the tube to the patient's elongated mustache [12].

### 3.7 Advantages of Innovative Fixation Methods

The included studies reported several clinically important advantages of the proposed fixation techniques. Primarily, these methods avoid jugular vein compression, thereby mitigating the risk of venous return impairment [9, 12, 13, 14, 15, 16, 17]. Additionally, the elimination of adhesive tapes reduces the risk of allergic reactions [12, 14]. These approaches also provide better surgical access during neurosurgical and facial procedures, making them particularly suitable for such interventions [9, 14, 16, 17]. Their broad availability and cost‐effectiveness were also emphasized [9, 14, 15]. In addition, the facilitation of facial skin integrity monitoring was highlighted as a principal benefit across multiple reports [15, 17].

### 3.8 Complications and Limitations of Innovative Fixation Methods

Thematic analysis within this domain identified three primary subthemes: (1) limited generalizability attributable to anecdotal evidence bases; (2) paucity of comparative evaluations against conventional fixation modalities; and (3) inadequate delineation of applicability across diverse surgical positions and prolonged intubation scenarios. The first two subthemes predominantly arise from the preponderance of case reports among the included studies [9, 12, 14, 15, 16, 17].

### 3.9 Comparative Synthesis

Overall, the reporting of results was heterogeneous and lacked standardized criteria, which precluded direct comparisons between techniques. However, the reviewed studies were limited by the inability to use standardized methods that could secure the endotracheal tube in bearded patients without interfering with the surgical field and focused primarily on tube stability, safety, and clinical utility. Most techniques were associated with adequate endotracheal tube fixation and low rates of accidental tube displacement or removal. However, these findings were largely anecdotal. Cover‐based techniques, such as the use of Tegaderm or synthetic covers, were relatively simple and preserved facial hair while improving adhesive performance. However, they required careful application and had not been consistently evaluated in long‐term procedures. Remote fixation methods had the advantage of not interfering with the surgical field, making them particularly suitable for neurosurgical procedures and prone positioning. However, they sometimes required additional equipment and preparation. They also avoided facial contact and jugular vein compression, as with the other two methods, and lacked skin sensitivity due to the absence of adhesive tape. Whisker‐based fixation was a simple and resource‐efficient approach, but it was limited to patients with sufficient facial hair length and was not applicable in all surgical settings. Overall, while all techniques aimed to improve tube stability without shaving, several studies also highlighted the advantage of avoiding jugular vein compression and lack of skin sensitivity due to the absence of adhesive tape. Complications have been rarely reported, but include potential risks related to specific limitations of the technique, such as difficulty in use or limited use in certain surgical situations. The use of these techniques in non‐recumbent positions and during prolonged surgical procedures has been inconsistently studied.

## 4 Discussion

This scoping review focuses on strategies for securing endotracheal tubes (ETTs) in patients with facial hair. This issue is often overlooked and not addressed in current guidelines from major organizations, including the American Society of Anesthesiologists (ASA). The main finding reveals that conventional adhesive tapes are ineffective for securing ETTs in this population. Additionally, traditional methods like neck bandaging are not recommended because they pose a risk of jugular vein compression, which can lead to hemodynamic instability and increased intracranial pressure. As a result, in the absence of specific guidelines for addressing these challenges, healthcare professionals have sought innovative solutions to ensure the security of endotracheal tubes without shaving patients' beards or interfering with the surgical field.

Most of the selected studies from India (*n* = 5) highlight the sociocultural context of the region, where the maintenance of facial hair is closely tied to religious and cultural beliefs. These beliefs often discourage individuals from shaving their facial hair, presenting unique challenges for healthcare providers during procedures that require the secure placement of endotracheal tubes [17]. As a result, there is a need for specific innovations that can preserve patient autonomy while ensuring effective fixation of the endotracheal tube.

Healthcare professionals employed three primary methods to tackle the outlined challenge: creating a covering for the patient's face, using remote fixation techniques, and fixing the ETT with the patient's mustache. The key advantages of these methods include maintaining tube stability without the need for shaving, avoiding pressure on the jugular vein, minimizing allergic reactions through limited skin contact, and no interference with the surgical field [14]. Some techniques also facilitate continuous monitoring of facial hair integrity, which is often an overlooked yet clinically significant aspect [17].

Minonishi et al. reported that changing patients from the supine to the prone position was associated with endotracheal tube displacement in approximately 92% of cases [18]. This highlights an increased risk of extubation when patients are in non‐supine positions. Notably, only two studies have demonstrated that this innovative fixation method can be applied safely in positions other than supine [9, 16], whereas the remaining studies did not address or consider this issue.

Similarly, Cheon et al. identified that prolonged intubation duration is an independent risk factor for endotracheal tube displacement and inadvertent extubation [19]. Despite this considerable finding, only one study has examined the use of this technique in surgeries lasting longer than 2 h [13]. The other studies did not specify the time parameters for implementing these innovative strategies. This lack of evidence in the literature raises legitimate concerns about the applicability of these methods for longer surgical interventions.

A major strength of the present review is its broad time range, covering articles published between 1990 and June 2025. This review also included a heterogeneous array of study designs, such as editorials, case reports, case series, letters to the editor, commentaries, narrative reviews, original research articles, and randomized clinical trials. This methodological diversity facilitates a more holistic apprehension of ETT fixation techniques and their challenges.

The main limitation of this review is the generally low methodological rigor of the included studies, which stems from their inherent design features. Only one study utilized clinical trial design, while the rest of the available literature consists solely of case reports detailing various fixation methods. Nonetheless, in accordance with the objectives of this study, all available articles were gathered to provide a comprehensive summary of endotracheal tube fixation methods for patients with facial hair. Additionally, the small number of included studies may indicate a broader lack of attention within the medical community regarding the importance of endotracheal tube placement in this patient population.

Several potential sources of bias should be considered when interpreting the findings of this review. The first is publication bias, which is likely to overreport successful or novel techniques. Second, most patients were not randomly selected, and a significant portion of the available evidence is derived from case reports and letters to the editor, which inherently carry a high risk of bias. These study designs lack control groups, randomization, and standardized outcome assessments, with incomplete reporting and shortcomings that can be attributed to noncompliance with reporting standards. The third is that most of the studies reviewed had small sample sizes, often limited to single patient reports, which reduces the generalizability of these methods. Finally, the lack of standardized protocols and outcome definitions across studies made comparisons difficult and prevented firm conclusions about the effectiveness of the reported techniques.

Therefore, we suggest that to ensure the scientific validity of innovative techniques and increase their generalizability, these approaches should be evaluated through randomized controlled clinical trials conducted in diverse patient populations and clinical settings. Such studies would allow for rigorous comparison of the efficacy and safety of these methods with current standard practices. Furthermore, we recommend that anesthesia professional organizations, such as the American Society of Anesthesiologists (ASA), systematically review the existing literature on endotracheal tube fixation in patients with facial hair and develop clear, evidence‐based guidelines to support clinicians in addressing this challenge.

## 5 Conclusion

Securing endotracheal tubes in patients with facial hair who refuse to shave remains a significant clinical challenge. Conventional fixation techniques, such as adhesive tape and neck bandages, are often ineffective due to reduced adhesion in the presence of facial hair. In addition, these methods may be associated with potential complications, including jugular vein compression and interference with the surgical field.

This scoping review identified seven studies describing innovative approaches, which can be broadly categorized into three strategies: facial covering techniques to improve adhesion, remote or indirect fixation methods that bypass facial hair, and mustache‐based stabilization techniques. These strategies offer several potential advantages, including preservation of facial hair, avoidance of venous compression, and reduced interference with surgical access. However, the current evidence base remains limited, as most reports consist of case reports without direct comparisons to standard techniques, thereby limiting generalizability. Future well‐designed clinical trials are warranted to evaluate the safety, effectiveness, and broader applicability of these innovative methods, with particular emphasis on the development of more advanced and clinically applicable solutions.

## Author Contributions

**Shaqayeq Taghizadeh:** conceptualization, writing – review and editing, writing – original draft, data curation, methodology, investigation. **Kimia Khonakdar:** data curation, validation, writing – original draft, writing – review and editing, visualization. **Shaghayegh Rezaeekia:** data curation, investigation. **Seyed Abolfazl Hosseini:** data curation, investigation. **Alireza Babajani:** conceptualization, methodology, data curation, writing – review and editing, project administration, supervision, writing – original draft, investigation.

## Funding

The authors have nothing to report.

## Ethics Statement

The authors have nothing to report.

## Consent

The authors have nothing to report.

## Conflicts of Interest

The authors declare no conflicts of interest.

## Transparency Statement

Alireza Babajani affirms that this manuscript is an honest, accurate, and transparent account of the study being reported; that no important aspects of the study have been omitted; and that any discrepancies from the study as planned (and, if relevant, registered) have been explained.

## Supporting information

Supporting File 1:

Supporting File 2:

Supporting File 3:

## Data Availability

Data sharing is not applicable to this article, as no new data were created or analyzed in this study.
