# Supplementary material for: Innovative Techniques and Challenges in Securing Endotracheal Tubes Among Patients With Facial Hair: A Scoping Review
Source: Health Sci Rep. 2026 Jun 30;9(7):e72721. doi: 10.1002/hsr2.72721 (PMC13316945; doi:10.1002/hsr2.72721)
Supplement: Supplementary file 2 — Supporting File 2: [file HSR2-9-e72721-s002.pdf]

Structured search strategies used in major databases	
Databases	search strategies
PubMed	("Way"[Title/Abstract] OR "Method"[Title/Abstract] OR "Procedure"[Title/Abstract] OR "Technique"[Title/Abstract] OR "Approach"[Title/Abstract]) AND ("Secure"[Title/Abstract] OR "hold*"[Title/Abstract] OR "Securing"[Title/Abstract] OR "stabilization"[Title/Abstract] OR "stable"[Title/Abstract] OR "fix"[Title/Abstract] OR "fixation"[Title/Abstract]) AND ("intratracheal intubation*"[Title/Abstract] OR "endotracheal intubation*"[Title/Abstract] OR "Endotracheal Tube"[Title/Abstract]) AND ("facial hair"[Title/Abstract] OR "beard*"[Title/Abstract] OR "moustache"[Title/Abstract] OR "mustache"[Title/Abstract] OR "hirsutism"[Title/Abstract])
Web of Science	TS=("Way" OR "Method" OR "Procedure" OR "Technique" OR "Approach") AND TS=("Secure" OR hold* OR "Securing" OR "stabilization" OR "stable" OR fix OR fixation) AND TS=("intratracheal intubation*" OR "endotracheal intubation*" OR "Endotracheal Tube") AND TS=("facial hair" OR beard* OR moustache OR mustache OR hirsutism)
Science Direct	TITLE-ABSTR("Way" OR "Method" OR "Procedure" OR "Technique" OR "Approach")<br>AND TITLE-ABSTR("Secure" OR hold* OR "Securing" OR "stabilization" OR "stable" OR fix OR fixation) AND TITLE-ABSTR ("intratracheal intubation*" OR "endotracheal intubation*" OR "Endotracheal Tube") AND TITLE-ABSTR("facial hair" OR beard* OR moustache OR mustache OR hirsutism)
Scopus	TITLE-ABS-KEY("Way" OR "Method" OR "Procedure" OR "Technique" OR "Approach")<br>AND TITLE-ABS-KEY("Secure" OR hold* OR "Securing" OR "stabilization" OR "stable" OR fix OR fixation) AND TITLE-ABS-KEY("intratracheal intubation*" OR "endotracheal intubation*" OR "Endotracheal Tube") AND TITLE-ABS-KEY("facial hair" OR beard* OR moustache OR mustache OR hirsutism))
Cochrane Library	(('Way' OR 'Method' OR 'Procedure' OR 'Technique' OR 'Approach'):ti,ab) AND (('Secure' OR hold* OR 'Securing' OR 'stabilization' OR 'stable' OR fix OR fixation):ti,ab) AND(('intratracheal intubation*' OR 'endotracheal intubation*' OR 'Endotracheal Tube'):ti,ab) AND(('facial hair' OR beard* OR moustache OR mustache OR hirsutism):ti,ab)
Embase	(('Way' OR 'Method' OR 'Procedure' OR 'Technique' OR 'Approach'):ti,ab) AND (('Secure' OR hold* OR 'Securing' OR 'stabilization' OR 'stable' OR fix OR fixation):ti,ab) AND(('intratracheal intubation*' OR 'endotracheal intubation*' OR 'Endotracheal Tube'):ti,ab) AND(('facial hair' OR beard* OR moustache OR mustache OR hirsutism):ti,ab)
