# Supplementary material for: Innovative Techniques and Challenges in Securing Endotracheal Tubes Among Patients With Facial Hair: A Scoping Review
Source: Health Sci Rep. 2026 Jun 30;9(7):e72721. doi: 10.1002/hsr2.72721 (PMC13316945; doi:10.1002/hsr2.72721)
Supplement: Supplementary file 3 — Supporting File 3: [file HSR2-9-e72721-s003.pdf]

Critical appraisal of included studies using JBI checklists and ROB 2 tool

Table 1. Risk of Bias Assessment for Randomized Controlled Trial

Study	D1	D2	D3	D4	D5	Overall
Kamalipour et al. 2003, Iran	SC	SC	SC	LR	SC	SC

D1: Randomization – Bias arising from the randomization process.
D2: Deviations – Bias due to deviations from intended interventions.
D3: Missing Data – Bias due to missing outcome data.
D4: Measurement – Bias in measurement of outcomes.
D5: Selection – Bias in the selection of the reported result.

Judgment:
LR: Low risk
SC: Some concerns
HR: High risk

Table 2. Joanna Briggs Institute Critical Appraisal for Case Reports

Study	Q1	Q2	Q3	Q4	Q5	Q6	Q7	Q8
Khorasani et al. 1996, United States	No	No	No	No	Yes	Unclear	No	Yes
Hooda et al. 2010, India	Yes	No	Yes	No	Yes	Unclear	No	Yes
Agarwal et al. 2011, India	Yes	No	Yes	No	Yes	Unclear	No	Yes
Kajal et al. 2015, India	Yes	No	Yes	No	Yes	Unclear	No	Yes
Brahma et al. 2020, India	Yes	No	Yes	No	Yes	Unclear	No	Yes
Singh et al. 2022, India	Yes	No	Yes	No	Yes	Yes	No	Yes

Q1: Were patient's demographic characteristics clearly described?
Q2: Was the patient's history clearly described and presented as a timeline?
Q3: Was the current clinical condition of the patient on presentation clearly described?
Q4: Were diagnostic tests or assessment methods and the results clearly described?
Q5: Was the intervention(s) or treatment procedure(s) clearly described?
Q6: Was the post-intervention clinical condition clearly described?
Q7: Were adverse events (harms) or unanticipated events identified and described?
Q8: Does the case report provide takeaway lessons?
